# How to help countries improve resilience during a pandemic: an example of a Rapid Exchange Forum

**DOI:** 10.1093/eurpub/ckae035

**Published:** 2024-07-01

**Authors:** Claudia Habl, Johannes Weiss, Anita Gottlob, Miriam Saso, Nienke Schutte, Petronille Bogaert, Marília Silva Paulo, Luís Velez Lapão

**Affiliations:** International Affairs, Policy, Evaluation and Digitialisation, Gesundheit Österreich GmbH, Vienna, Austria; International Affairs, Policy, Evaluation and Digitialisation, Gesundheit Österreich GmbH, Vienna, Austria; International Affairs, Policy, Evaluation and Digitialisation, Gesundheit Österreich GmbH, Vienna, Austria; EU Health Information System Department, Sciensano, Brussels, Belgium; EU Health Information System Department, Sciensano, Brussels, Belgium; EU Health Information System Department, Sciensano, Brussels, Belgium; CHRC, NOVA Medical School, Faculdade de Ciências Médicas, NMS, FCM, Universidade Nova de Lisboa, Lisbon, Portugal; CHRC, NOVA Medical School, Faculdade de Ciências Médicas, NMS, FCM, Universidade Nova de Lisboa, Lisbon, Portugal; UNIDEMI, NOVA FCT, Universidade Nova de Lisboa, Lisbon, Portugal; LASI Intelligente Systems Associated Laboratory, Guimarães, Portugal

## Abstract

**Background:**

The COVID-19 pandemic demanded quick exchanges between experts and institutions supporting governments to provide evidence-based information in response to the crisis. Initially, there was no regular cross-country forum in the field of population health. This paper describes the set-up and benefits of implementing such a forum.

**Methods:**

A group of public health practitioners from academia, national public health institutes and ministries of health decided in April 2020 to meet bi-monthly to discuss a vast array of population health topics in a structured format called a Rapid Exchange Forum (REF). An ad-hoc mailing group was established to collect responses to questions brought forward in the forum from at least five countries within 24 h. This endeavour, which evolved as network of networks was awarded an EU grant in autumn 2020 and was called PHIRI (Population Health Information Research Infrastructure).

**Results:**

Responses from up to 31 countries were compiled and shared immediately via the European Health Information Portal. This exchange was complemented by special REFs that focused on the advantages and disadvantages of vaccination, for example. By July 2023, 54 REFs had taken place with topics going beyond COVID-19.

**Conclusion:**

The REF demonstrated its value for quick yet evidence-based cross-country exchange in times of crisis and was highly appreciated by countries and European Commission. It demonstrated its sustainability even after the acute crisis by expanding the topics covered and managing to continue exchange with the aim of capacity building and mutual learning, making it a true EU response and coordination mechanism.

## Introduction

The Population Health Information Research Infrastructure (PHIRI) was developed to facilitate the exchange and generation of the best available evidence for decision makers and researchers regarding the health of populations impacted by COVID-19. PHIRI consists of experts from 41 partner organizations in 30 countries who collaborated to set-up the joint European Health Information Portal (www.healthinformationportal.eu).^1^ Another objective of PHIRI was to address the challenges of health information research related to COVID-19, thus taking responsibility for dynamizing European public health institutes and research centres to improve communication and the sharing of experiences.

The COVID-19 pandemic took most countries by surprise and demanded exchange of knowledge, insights, and data between experts and institutions supporting national governments in response to the crisis from the very first day. The dynamic nature of the virus, the scarce scientific evidence available, and the impact of the virus on the population required swift coordination and collaboration to implement timely and appropriate measures.

To address this need for quick, yet trustworthy and evidence-based exchange, the current PHIRI partners (who partly had worked together in other health information projects, e.g. the Joint Action InfAct^2^) started already in March 2020 to organize meetings to ensure knowledge exchange and transfer of evidence between countries.

This forum for regular cross-country exchange represented a unique format in the field of population health despite many, very topic-specific, expert groups were established to combat the crisis. The first meeting on 6 April 2020 was no stand-alone exchange between researchers but involved staff from public health institutes, ministries of health, regional authorities and European Commission services engaged in crisis control and containment.

The major aim of the meetings, named Rapid Exchange Forums (REFs), was to offer a true Europe-wide response and coordination mechanism, covering a wide area of topics regarding the health and well-being of populations impacted by COVID-19. The attendees agreed to retain their focus on population health and build up a network with other institutions and networks. These included bodies like the Joint Research Committee (JRC), national crisis response teams, and the EU’s Health Security Committee that was set-up in 2001 as an informal advisory group on health security at European level.^20^

Once PHIRI was launched in autumn 2020, the REF became a bi-weekly meeting that brought together experts across Europe to exchange the most up-to-date evidence on whatever topic was under discussion, soon evolving in a network of networks. Together with 12 other EU-funded projects with a combined funding of €79 million, PHIRI created the PREparedness and resPonse for emergency situAtions in euRopE (PREPARE) cluster^3^ to avoid overlaps in activities, exchange learning and scale up results.

In early 2021, the cooperation was extended to the European Centre for Disease Prevention and Control (ECDC) in two ways: the Centre was invited to attend REF meetings and ask questions in the agreed format plus the REF helped monitor and display Public Health and Social Measures, leading to the establishment of the Corona Corner on the European Health Information Portal.

**Figure 1:**
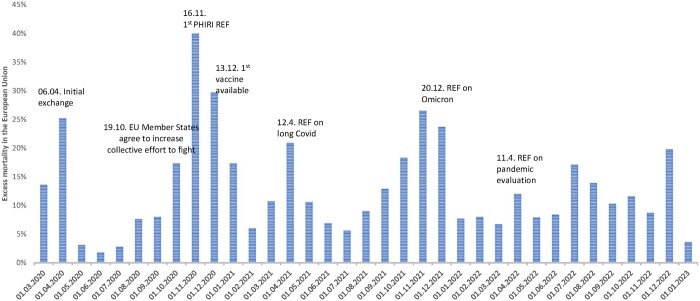
Excess mortality in EU member states from March 2020 to January 2023 and PHIRI Rapid Exchange Forum (REF) milestones. Sources: American Society for Microbiology,4 EUROSTAT.5.

The intention behind the REFs was to tackle COVID-19 topics that were either already high on the agenda of national decision makers or which the group deemed to be of imminent upcoming relevance. Figure 1 shows how selected REF topics linked to the evolution of excess mortality in the EU, where the mortality rates of each month were compared to the corresponding month of the average rates of 2016–2019.^4^^,^^5^

## Methods

This paper describes the methods and processes (documentation, topic selection, organization) used in the REF and then analyses especially the period between October 2020 and May 2023 with a focus on vaccination-related topics.

### Standard operating procedures and resources of REF

#### Governance

*Hosting and moderation:* The founders, the Austrian National Public Health Institute GÖG and the Belgian Sciensano acted as Secretariat of the REF. They prepared, organized, hosted and documented each REF where Sciensano had the lead in the special REF and GÖG in all others.

*Target audience:* Experts from public health institutes, crisis response teams, advisors to policy makers and researchers. Focus was put on PHIRI consortium members but representatives from other countries and crisis response bodies were invited as well.

#### Topic selection and documentation

*Structured collection of topics:* Questions from EU institutions and national crisis response teams were collected and put to the vote on a weekly basis organized using the Lime Survey tool. The top ranked questions were sent to REF members, who replied at the REF meeting or within two days after. In addition to the structured collection of questions, countries could submit ad-hoc queries in a structured format by email. The Secretariat distributed these queries, with the responsibility of compiling answers left to the country raising the question.

Due to the high demand in Q4/2020 the Secretariat made a needs assessment survey in Q1/2021 to prioritize the high number of incoming questions. This needs assessment survey identified current and future priorities of European countries regarding their pandemic management, and collected lessons learned including a rating of countries’ national COVID-19 pandemic management in specific predefined areas during the first waves.^6^

*Regular Meeting Schedule:* The REF sessions were scheduled bi-monthly, always using the same video link.

*Consistent Meeting Structure:* The REF sessions adhered to a strict agenda, starting with the session’s primary topic and pertinent announcements. The latest guidelines were presented, as were emerging research findings in the field. The core of each session was dedicated to insights and contributions from attending country experts, and if deemed necessary specialists for the chosen topics were invited, too.

*Publicity:* All replies to questions put forward in a REF and to ad-hoc queries were collected and made available on PHIRI’s intranet as well as on the European Health Information Portal.

#### Systematic exchange and gathering of evidence on international guidelines and policy measures

Each REF featured (i) the presentation of COVID-19 related international guidelines, projects and information sources and (ii) the monitoring of COVID-19 policy and impact measures. Once the REF topic was sent out, PHIRI partners would review international websites, seeking the latest updates on guidelines, consult their networks about new and upcoming projects, and search national health-related databases for scientific evidence. Priority was given to systematic reviews and meta-analyses that would pool together a set of different estimates, aiming to obtain the best evidence that is needed for the most informed decision making. Another relevant resource used was a set of interactive maps, dashboards, and catalogues sand lists of Public Health and Social Measures applied by country over time, as displayed on the Corona Corner of the European Health Information Portal.^7^

#### Compiling evidence on public health and social measures

The REFs revealed a need to compile evidence of Public Health and Social Measures and the impact of their health-related policies on the management of COVID-19 throughout the different stages of the pandemic. To accomplish this, we identified and selected scientific literature on Public Health and Social Measures to provide information on measures that could contribute to containing the spread of the SARS-CoV-2 virus. The framework used was the ‘Oxford COVID-19 Government Response Tracker’ indicator set developed by the Blavatnik School of Government.^8^ The concept of Public Health and Social Measures is used instead of non-pharmaceutical interventions (NPIs), as suggested by the World Health Organization (WHO), due to its clear and inclusive characteristics that describe public health and social interventions as ‘measures or actions by individuals, institutions, communities, local and national governments, and international bodies to slow or stop the spread of infectious diseases, such as COVID-19.^9^

#### Special REFs and webinars

In the context of the pandemic, it was crucial to be aware of ongoing COVID-19 activities and expert groups internationally to avoid the duplication of efforts and promote collaboration. In response to this need, the REF—Special Edition was launched, serving as a global collaboration platform, and convening every 3–6 months to bring together national and international experts to discuss existing and emerging activities and initiatives in response to the pandemic.

These events offered a valuable opportunity to exchange knowledge, identify opportunities for collaboration, and ensure alignment of international efforts in addressing the pandemic. Such meetings launched new collaborations, especially with the ECDC regarding the COVID-19 Policy Measures section on the European Health Information Portal^7^ and the ECDC-JRC Response Measures Database^10^ (now discontinued).

### Document analysis

We used document-analysis for this work and evaluated the participation and responses of participants to obtain the overall participation rate. Also, if more than one person per country respectively EU or international organization took part, this was counted as one participant or reply.

In a second step, a frequency analysis was performed (using R) to illustrate the frequency of topics in all REF meetings that took place between in the observation period. The analysis was based on an overview of the content that was discussed as extracted from the minutes of the meetings, the REF responses, and the REF topic list.^11^

Finally, we manually/qualitatively examined how issues evolved over time within a sub-sample of the most common REF topic, namely vaccination. To do so, we looked at the subtitles and content (from the minutes and titles of the meetings) within all vaccine-related meetings and clustered themes together. Finally, we analysed engagement within the vaccine topic using the participation rates and replies at or after each meeting.

## Results

### Overall participation rate

The PHIRI consortium includes 30 countries as partners, namely Austria, Belgium, Bosnia and Herzegovina, Bulgaria, Croatia, the Czech Republic, Estonia, Finland, France, Germany, Greece, Hungary, Ireland, Italy, Latvia, Lithuania, Luxembourg, Malta, Moldova, the Netherlands, Norway, Poland, Portugal, Romania, Serbia, Slovakia, Slovenia, Spain, Sweden and the United Kingdom.

The REF exchanges, i.e. attendance to meetings and/or replying to questions raised in the REFs or via ad-hoc query included all countries participating in PHIRI except for France and Luxembourg, plus the non-PHIRI partners Albania, Cyprus and Denmark making it altogether 31 countries giving input to one of the topics brought forward in REF meetings.

Additionally, 11 bodies resp. organizations, namely ECDC, European Public Health Association (EUPHA), Eurostat, European Observatory on Health Systems and Policies, Standing Committee of European Doctors, OECD, WHO, European Medicines Agency, JRC and representatives of General Directorates of the European Commission (e.g. from Directorate-General for Health and Food Safety (DG SANTE) or Directorate-General for Research and Innovation (DG RTD) or of the PREPARE^3^ cluster (e.g. ELIXIR) attended meetings. Attendance for meetings were free of charge and no ex-ante registration was necessary.

Responses were compiled and shared on a devoted website at the European Health Information Portal.

**Figure 2:**
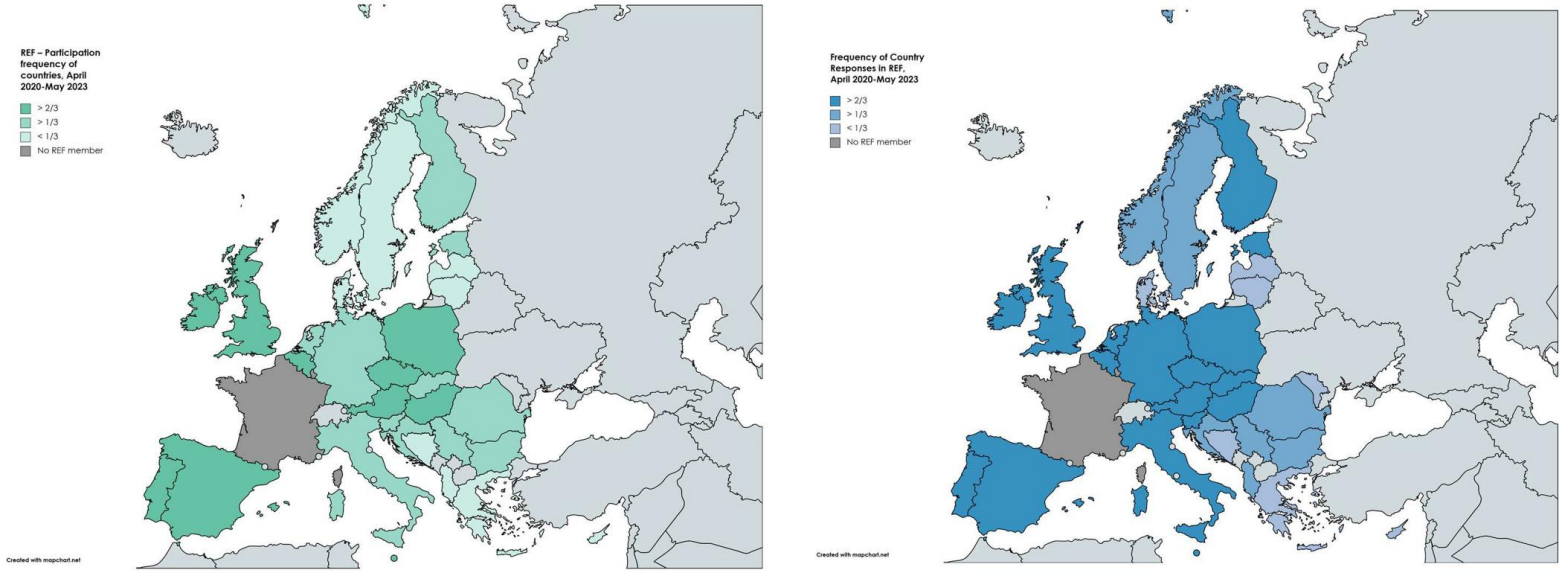
Participation of countries in REFs and frequency by which countries provide a reply to issues put forward in REF (country responses), April 2020 to May 2023 Source: GÖG-PHIRI Secretariat 2023.11.

The two maps in figure 2 show the level of interaction of the participants, both for attendance to REF meetings and inputs to questions brought forward in the REFs.

Austria and Belgium had the highest attendance rates due to hosting and facilitating the REF activities. These both countries, together with Hungary, Portugal, Italy, Ireland and Malta had the highest number of inputs.

### Recurring topics in REFs

**Figure 3:**
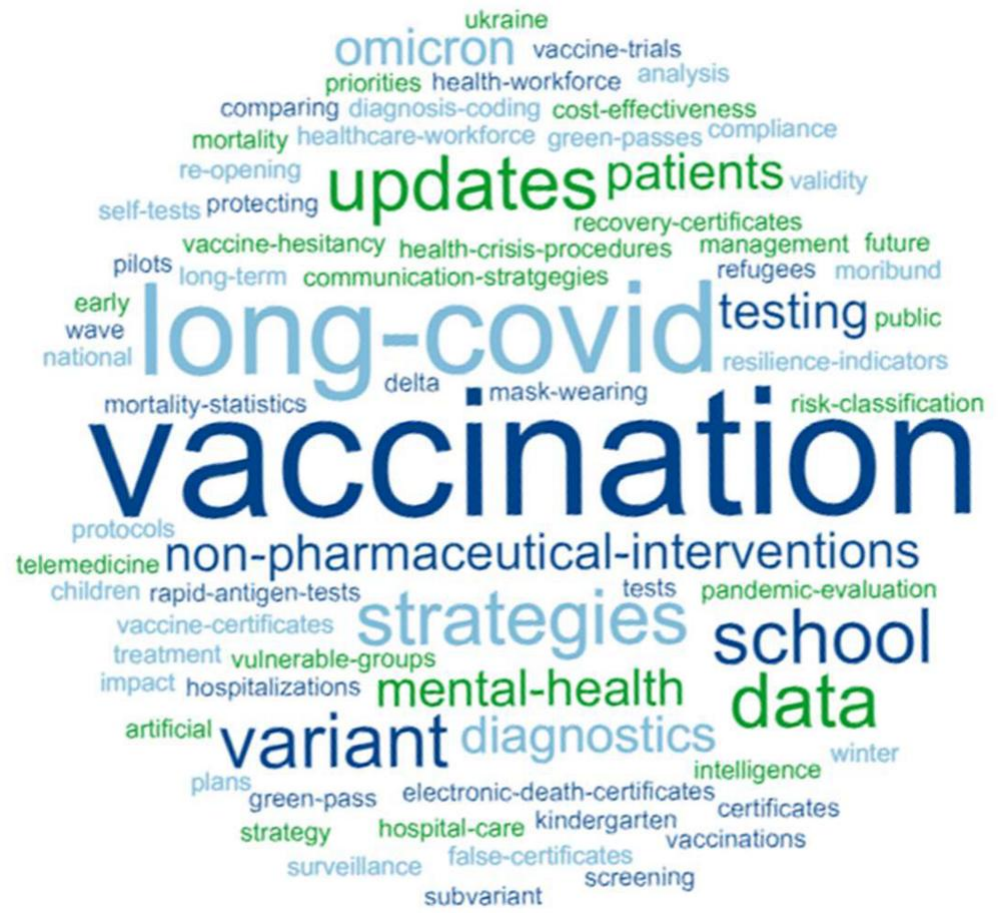
Word cloud on major topics in REFs November 2020 to May 2023 Source: GÖG-PHIRI Secretariat 2023.11.

By July 2023, 54 REF meetings had taken place, with topics going beyond COVID-19 after November 2022, such as the digitalization of health certificates or preparedness for future health emergencies. The word cloud in figure 3 highlights the reoccurrence of vaccination-related topics followed by long Covid, non-pharmaceutical interventions and mental health.

A keyword search of the topics covered in the REFs^4^ revealed that 25% (13 out of 53) of the exchanges (November 2020 to May 2023) either specifically focused on the topic of vaccination or included questions on vaccination, mostly in 2021. This coincided with the time when the pharmaceutical providers Moderna and AstraZeneca were first granted marketing authorization for EU-wide use, following the first COVID-19 vaccine by BioNTech/Pfizer in late December 2020.^8^^,^^12^^,^^13^

A subsequent analysis of reoccurring themes within a selected sample of REF exchanges on vaccination illustrates the overall development of related issues during the REF activities (November 2020 to October 2022). The results suggest that the earlier REFs on vaccination focused on issues such as vaccination strategies, planning, purchasing strategies and compliance. The first meeting, for instance, covered vaccination strategies (e.g. which age groups, prioritizing), mass testing strategies, and which vaccines were most likely to be used in each country. From mid-to-late 2021 and in 2022, novel issues such as vaccine hesitancy, false certificates or COVID-19 vaccination in children arose. Documentation shows that addressing public health needs was a key point of discussion, including questions on vaccine efficacy, vaccine development and specific population groups.

**Figure 4:**
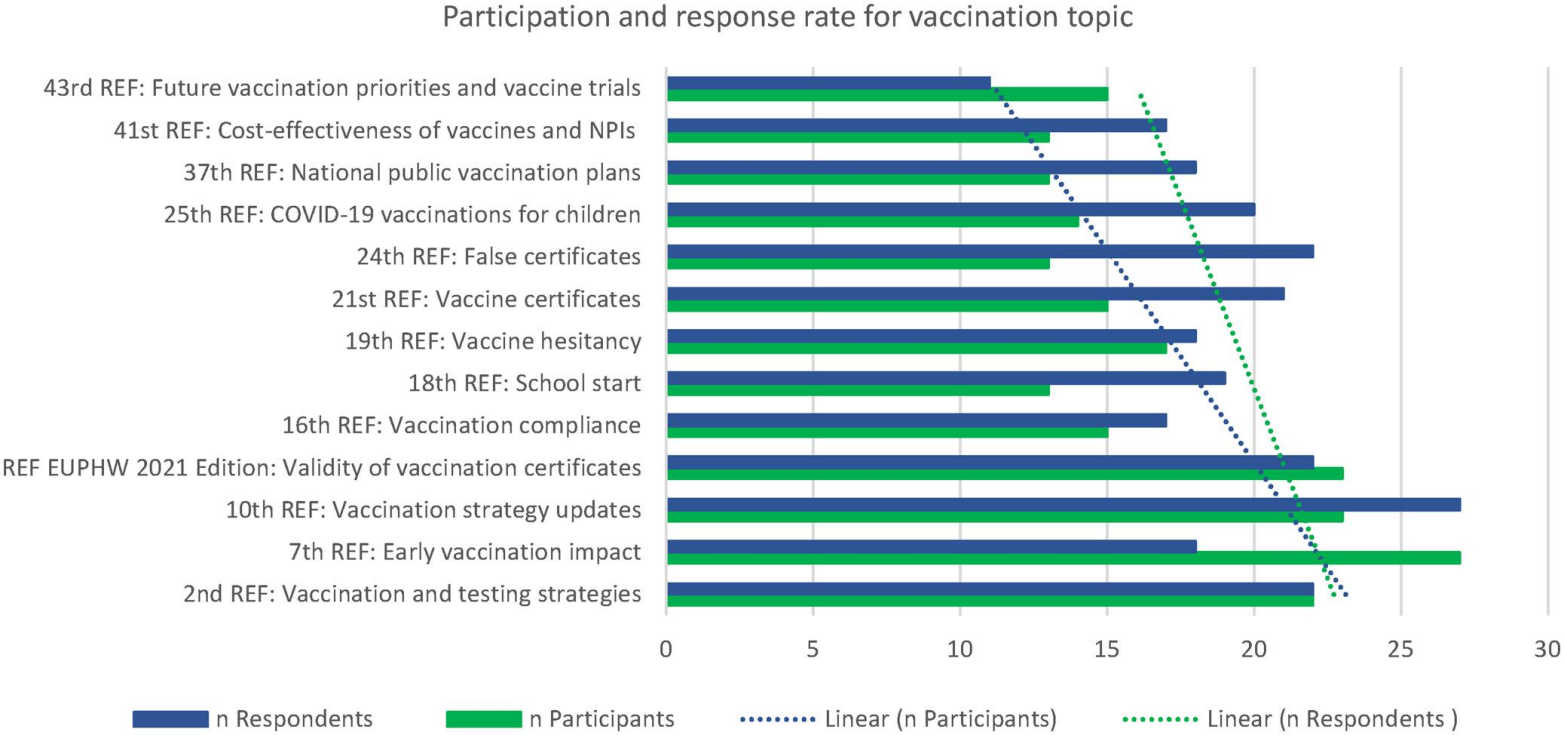
Participation and response rates on the topic of vaccination in REFs between November 2020 and October 2022 Source: GÖG-PHIRI Secretariat 2023.11.

Figure 4 shows that participation and the response rate were particularly high for early vaccination impact (66% participation rate) and vaccination strategies and updates on them.

Both, participation, and written response rates decreased over time for vaccination-related topics, namely by 32% and 50%, respectively.

## Discussion

The COVID-19 pandemic confronted countries with challenges that required immediate and continuous information exchange among experts and institutions within countries and across Europe. Having a pre-established network for broad, yet not too formal, information exchange and a coordinated response on urgent public health issues is of utmost importance as it can enable efficient and rapid communication among relevant stakeholders for any future crisis in health and care as the REF example showed. The REFs provided a platform for participants to openly share and discuss various COVID-19 topics in a trustworthy setting, allowing countries to learn from each other’s experiences and access the most up-to-date evidence available. Further, they enabled participating public health organizations to share and discuss the rationale behind the implementation of Public Health and Social Measures in each country and thereby had a direct impact on national decision making. For instance, face shields made of transparent plastic became popular in summer 2020. Doubts on the value of this protective measure arose in the field of public health. Alongside other evidence, in Austria, information on the use of these face shields as the sole protective measure in most countries collected in a REF exchange meeting played a decisive role in banning them in mid-October 2020. Similarly, early in REF history, a prominent focus was placed on the measurement of COVID-19 related deaths, revealing implementation disparities across countries despite an agreed EU standard. Notably, findings from the REF meeting were swiftly reported to the Health Security Committee and Eurostat.

Analysis suggests (see figure 4) that participation at REF meetings was especially high for issues relevant to policymakers such as vaccination strategy updates or the question of cross-country certificates. Addressing these issues in a timely manner to compare and discuss evidence from and the experiences of public health experts and organizations across countries facilitates long-term capacity building as well as mutual learning in this field.

Some of these latter issues, such as vaccination hesitancy, relate to public perceptions and attitudes towards vaccination that developed and/or were reinforced during the pandemic. Research suggests that COVID-19 vaccine hesitancy may have a spill-over effect onto how people view other vaccinations in general^14^^,^^15^^,^^21^ This could become a long-term public health issue and cause unforeseen or negative consequences in future health crises.^16^ Studies also show that COVID-19 vaccine hesitancy and uptake varied across EU countries^17^ with the effectiveness of interventions possibly varying depending on underlying motivations.^18^ This issue was also discussed in PHIRI sessions, for example as part of Infodemics at the 2022 EUPHA conference.^19^

The frequency and continuity of discussions among participants for over three years underline the significance of having a public network for information exchange and highlight the commitment to share knowledge across countries.

Another positive feature was the close cooperation with other networks (PREPARE cluster), EU services (JRC, ECDC) and the WHO, which allowed for stringency in generating evidence and ensured up-to-date information throughout, making PHIRI’s REFs an important instrument in the European toolbox to tackle COVID-19. This is reflected in a lecture at the EU crisis and response workshop of the Special Committee of the European Parliament on the COVID-19 pandemic in March 2023, which highlighted REF’s ability to anticipate upcoming needs and ability to share information in a trusted manner. A further asset, the Corona Corner of the European Health Information Portal,^7^ which offered a concise and timely overview of the most common Public Health and Social Measures at a given time in European countries is a good example for the close cooperation with the ECDC as it complemented their general provision of information on measures in place.^10^

Limitations of the REF include differing levels of approval for sharing information required by countries and organizations participating in the REF meetings, especially concerning the release of confidential information, as well as the diversity of bodies involved in providing a response within individual member states. This introduces complexities in both representation and perspectives. At the same time, these complexities enhance the overall credibility and validity of the REF meetings. Also, attendance rates by countries differed largely (e.g. the PHIRI consortium member France never contributed to REF as it focused on bilateral exchange) and showed a downward trend by the end of 2022.

Still, the usefulness of the REFs was again demonstrated when the pandemic started to fade and the decision was taken in November 2022 to expand the topics beyond COVID-19 and to continue capacity building, for example in the form of the first PHIRI spring school in March 2023.

In this light, we believe that the REF network and its resources, fortified by the trust it has built, are potent tools for fostering collaboration and addressing information needs, as opposed to merely making data available. Emphasizing the importance of trust and responsiveness, we advocate for a collaborative approach to enhance the collective readiness of EU member states in addressing future challenges. Together with support from policymakers, we can increase our resilience in public health.

In summary, the dynamic nature of the REFs underscores their profound value to the community of public health experts. The success of the REF meetings can be attributed to several key factors, notably a foundation of trust that fosters swift sharing of information among participants; an ability to anticipate upcoming information needs effectively; a broad spectrum of topics covered (in contrast to many very topic-specific networks), addressing diverse public health concerns; and PHIRI’s role as a network of networks, consistently directing queries to the most relevant experts within organizations.

Concluding, the REF meetings represent a unique and pivotal forum that embodies the spirit of collaboration, enabling us to adapt and respond to the ever-evolving landscape of public health. As we confront future crises, the REF stands as an indispensable tool for forging a united front in the face of uncertainty and new health emergencies.

## Data Availability

The data underlying this article are available from www.healthinformationportal.eu and from www.phiri.eu.

## Disclaimer

The content of this manuscript represents the views of the authors only and is their sole responsibility. The European Research Executive Agency (REA) and the European Commission are not responsible for any use that may be made of the information it contains.

Key points

- PHIRI’s activities are important building blocks in improving the resilience of Europe and its institutions dealing with public health to potential new crisis situations.
- The existence of the REF network has promoted numerous bilateral contacts between countries and international organizations and has facilitated the adoption of successful strategies from one context to another. This was also recognized by the European Commission and its services (e.g. ECDC).
- It is necessary to maintain both, the European Health Information Portal as a repository for evidence and findings and the REF exchange as an option for further mutual learning and capacity building, including a regular spring school on health information.
- The PHIRI group is preparing a sustainability plan to carry on with the REF meetings and the Health Information Portal to formalize and guarantee this channel of communication for both public administration and researchers and calls policy makers to support this existing EU response and coordination mechanism.

## Notes

On behalf of the Population Health Information Research Infrastructure.
